# Increasing chromosome 1 copy number parallels histological progression in breast carcinogenesis

**DOI:** 10.1054/bjoc.1999.1064

**Published:** 2000-02-21

**Authors:** M C Cummings, M Aubele, A Mattis, D Purdie, P Hutzler, H Höfler, M Werner

**Affiliations:** Department of Pathology, University of Queensland Medical School, Herston, Queensland, 4006, Australia; Institute of Pathology, GSF-Forschungszentrum für Umwelt und Gesundheit, GmbH Ingolstädter Landstrasse 1, Neuherberg, D-85764, Germany; Institut für Allgemeine Pathologie und Pathologische Anatomie, Der Technischen Universität München, Klinikum rechts der Isar, Ismaninger Str. 22, München, 81675, Germany; Department of Social and Preventive Medicine, University of Queensland Medical School, Herston, Queensland, 4006, Australia

**Keywords:** chromosome 1, FISH, breast carcinoma, histopathology

## Abstract

Chromosome 1 copy number in the benign breast lesions hyperplasia and atypical duct hyperplasia (ADH) was investigated using fluorescence in situ hybridization on paraffin sections. Progression of chromosome 1 changes occurring in parallel with histological progression from normal through hyperplasia and ADH to ductal carcinoma in situ (DCIS) and invasive carcinoma was also assessed, both overall and within individual patients. The mean signal number for normal cells was 1.14, while that for hyperplasia was 1.56 and ADH was 1.5, while values for DCIS of 1.95 and invasive duct carcinoma of 1.79, were higher (*P*< 0.001). Six of the seven cases also showed a significant trend towards an increasing proportion of cells with greater than 2 signals per nucleus occurring with histological progression (*P*< 0.001). These results support the concept that benign proliferative breast disease is a biological precursor of in-situ and invasive ductal carcinoma, the early histological changes possibly indicating a field effect with further genetic changes required for the development of a malignant phenotype. © 2000 Cancer Research Campaign


*British Journal of Cancer* (2000) **82**(6), 1204–1210
© 2000 Cancer Research Campaign
Article no. bjoc.1999.1064, available online at http://www.idealibrary.com on IDEAL®

### Increasing chromosome 1 copy number parallels histological progression in breast carcinogenesis

**MC Cummings[1], M Aubele[2], A Mattis[3], D Purdie[4], P Hutzler[2], H Höfler[2,3] and M Werner[3]**

[1]Department of Pathology, University of Queensland Medical School, Herston, Queensland, Australia 4006; [2]Institute of Pathology, GSF-Forschungszentrum für Umwelt und Gesundheit, GmbH Ingolstädter Landstrasse 1, D-85764 Neuherberg, Germany; [3]Institut für Allgemeine Pathologie und Pathologische Anatomie, Der Technischen Universität München, Klinikum rechts der Isar, Ismaninger Str. 22, 81675 München, Germany; [4]Department of Social and Preventive Medicine, University of Queensland Medical School, Herston, Queensland, Australia 4006

**Summary** Chromosome 1 copy number in the benign breast lesions hyperplasia and atypical duct hyperplasia (ADH) was investigated using fluorescence in situ hybridization on paraffin sections. Progression of chromosome 1 changes occurring in parallel with histological progression from normal through hyperplasia and ADH to ductal carcinoma in situ (DCIS) and invasive carcinoma was also assessed, both overall and within individual patients. The mean signal number for normal cells was 1.14, while that for hyperplasia was 1.56 and ADH was 1.5, while values for DCIS of 1.95 and invasive duct carcinoma of 1.79, were higher ($P < 0.001$). Six of the seven cases also showed a significant trend towards an increasing proportion of cells with greater than 2 signals per nucleus occurring with histological progression ($P < 0.001$). These results support the concept that benign proliferative breast disease is a biological precursor of in-situ and invasive ductal carcinoma, the early histological changes possibly indicating a field effect with further genetic changes required for the development of a malignant phenotype. © 2000 Cancer Research Campaign

**Keywords:** chromosome 1; FISH; breast carcinoma; histopathology

It is generally accepted that invasive ductal carcinoma of the breast arises from pre-existing ductal carcinoma in situ (DCIS) and, reflecting this link, the grade of the invasive cancer often parallels that of the associated in-situ component. Whether or not ductal epithelial hyperplasia and atypical ductal hyperplasia (ADH) are direct precursors of DCIS is less clear.

Many adenocarcinomas of the endometrium arise on a background of endometrial hyperplasia with and without atypia (Fox and Buckley, 1982). In the cervix, squamous cell carcinoma develops from an initially low grade and later high grade squamous dysplasia before developing into in-situ and then invasive malignancy (Buckley et al, 1982). Evidence for comparable disease progression in the breast is largely based on epidemiological studies. DCIS left untreated, is associated with a tenfold risk of subsequently developing invasive carcinoma and that occurs in the same area of the breast. Duct epithelial hyperplasia and ADH are associated with 1.5- and 4.6-fold increased risk respectively of later invasive malignancy, but in contrast to DCIS, that later invasive disease can occur anywhere in either breast (Page and Dupont, 1990). Hyperplasia and ADH of the breast thus appear to be markers of an increased risk of invasive disease, rather than obligate precursors, as is the case with DCIS.

Some recent studies have shown abnormalities occurring early in the possible pathway of breast cancer evolution. Loss of heterozygosity (LOH) has been demonstrated at a number of loci in both hyperplasia and ADH (O'Connell et al, 1994; Lakhani et al, 1995, 1996). This implies that at least some hyperplasias are clonal or neoplastic proliferations.

Abnormalities of chromosome 1 are one of the commonest chromosomal abnormalities to occur in invasive breast cancer, demonstrated in up to 80% of cases, often with three or more copies of the chromosome present (Heim and Mitelman, 1995). Increased chromosome 1 copy number is not just a late change occurring in aneuploid tumours, nor does it just occur in invasive carcinomas. Harrison et al (1997) demonstrated that six of eight cases of invasive breast cancer with chromosome 1 aneusomy were aneuploid and conversely, that six of another eight cases with a normal chromosome 1 complement were aneuploid. Earlier, Harrison et al (1995) had shown that 18 of 21 cases of DCIS had increased chromosome 1 copy number. These cases were predominantly of high nuclear grade or showed comedo necrosis.

We sought to investigate whether chromosome 1 aneusomy occurs in ductal epithelial hyperplasia and ADH and thus whether it could have an early causal role in the development of breast cancer. We also wished to see if there was a progression of chromosome 1 abnormalities that paralleled the histological progression from normal, through hyperplasia and ADH to in-situ and invasive disease, both overall and within individual patients.

### MATERIALS AND METHODS

### Tissue samples

Formalin-fixed, paraffin-embedded blocks from seven cases which included both proliferative breast disease and carcinoma, were selected from the files of the Institute of Pathology,

*Received 14 July 1999*
*Revised 16 September 1999*
*Accepted 18 October 1999*

*Correspondence to:* MC Cummings

Klinikum rechts der Isar. Tissue was diagnosed as normal, or showing duct epithelial hyperplasia, ADH, DCIS or invasive carcinoma. The criteria for distinguishing hyperplasia and ADH were according to those of Page and Anderson (1987). Hyperplastic foci showed increased cells, with disordered placement, indistinct cell borders, irregular secondary spaces and varied nuclear features. Classifying a lesion as ADH is notoriously difficult and there is significant interobserver variation. The criteria used included groups of cells in which there was a suggestion of sharply punched out secondary spaces but these were not completely developed through the entire primary space. The cells also showed more uniform placement, together with hyperchromasia and generally single small nucleoli. DCIS was subclassified into low, intermediate and high nuclear grade according to the criteria of Schwartz et al (1997). The modified Bloom and Richardson grading scheme of Nottingham was used for grading the invasive ductal carcinoma (Elston, 1987).

While precisely defining a lesion as ADH may not always be realistic, for practical purposes within this study, ADH lesions had histological features intermediate between those of hyperplasia of usual type and unequivocal DCIS. They were intermediate in terms of nuclear placement, definition of cell boundaries and shape of secondary spaces, as well as cytonuclear features.

Regions were assigned to histopathological categories by two pathologists (MCC and MW) using a conference microscope. Normal breast tissue was present in each block selected and this was used as an internal control for the variation in signal number that may occur with slight differences in section thickness. Consecutive 5-μm serial sections were cut for haematoxylin and eosin staining (H&E) or left unstained for fluorescence in situ hybridization (FISH).

### DNA probe

The probe CEP1, specific for the centromeric region of chromosome 1 (1q12 Spectrum Orange, Vysis, Stuttgart, Germany) was used. The specificity of the probe was confirmed on metaphase preparations from peripheral lymphocytes from a healthy donor.

### Slide preparation and hybridization

FISH reactions were prepared with slight modifications of established protocols (Zitzelsberger et al, 1994; Aubele et al, 1997). The predigestion stages included: 70% formic acid for 15 min, incubation in 1 M NaSCN at 80°C, for 10 min, and Pronase digestion. Five hundred microlitres of 0.5 mg ml$^{-1}$ Pronase E in phosphate-buffered saline (PBS) was applied to each tissue section, maintained on a warming box at 37°C, for approximately 5–7 min (range 3–12 minutes). Each section was viewed periodically using phase-contrast microscopy to assess the nuclear features of the unstained sections. Tissue digestion was considered complete when the nuclear outlines became crisply defined. Digestion was stopped by rinsing the slides in PBS, followed by incubation in glycine (2 mg ml$^{-1}$ in PBS) for 30 min at room temperature. One microlitre of CEP 1 probe (Vysis, Germany), 2 μl of purified water and 7 μl of CEP hybridization buffer was used in hybridization. Post-hybridization washing was performed at 43°C as previously described (Zitzelsberger et al, 1994). Nuclei were counterstained with 1 μg ml$^{-1}$ 4′,6-diamidino-2-phenylindole (DAPI) in antifade solution.

### Image acquisition

For FISH analysis fields of view were selected comparing hybridized and counterstained slides with immediately adjacent H&E-stained serial sections allowing unambiguous assignment of cells to specific histological categories. Fields of view were scanned with a confocal laser scanning microscope LSM 410 (Carl Zeiss, Jena) and a 100 × objective (Zeiss, PNF, NA 1.3, oil immersion) was used. A sequence of confocal optical sections was taken at axial distance of 0.5 μm, covering the full thickness of the histological section (Aubele et al, 1997). The FISH signals labelled with spectrum orange were taken using excitation at 543 nm and emission greater than 590 nm. The DAPI counterstain of the nuclei was excited at 364 nm and detected within the spectral range 400–430 nm. A third fluorescence channel (excitation 488 nm, emission 515–565) was used to identify non-specific background. Extended depth of view images were calculated from the image sequences by maximum projection. Finally the resulting images were overlayed on an RGB-display for signal counting.

### Evaluation

Signal counting was performed by one person (MCC). Only lesional cell nuclei were included and overlapping nuclei were excluded. In addition, only signals of a similar size and intensity were counted. Different entities were all geographically distinct and were often located in different blocks. Normal fields were assessed from each block to provide a control for possible variations in section thickness. The mean signal number per cell was calculated by dividing the total number of hybridization signals by the total number of nuclei counted. This gave an average chromosomal copy number for each histological entity and was best suited to describe clonal changes overall. The signal distribution or the percentage of cells with 0, 1, 2 or more than 2 signals per nucleus was also determined. Nuclear truncation due to tissue sectioning results in signal losses, making assessment of monosomy difficult. Evaluating normal breast ducts and lobules in each tissue section allowed chromosome under-representation to be defined (for this study) as signal numbers significantly less than that demonstrated for the corresponding normal tissue of that same section from that same block.

### Statistical analysis

Mean signal number was compared across the various tissue types (normal and abnormal) using one-way analysis of variance (ANOVA). The mean signal number for abnormal tissue was also examined relative to the mean number in the normal tissue from the same section (‘normalized’ means) to adjust for differences in section thickness. Multivariate regression techniques were also used to compare mean signal number in abnormal tissue, after adjusting for the mean numbers in normal tissue from each section (to adjust for section thickness). The Mantel–Haenszel extension to the $\chi^2$ statistic (Mantel and Haenszel, 1959) was used as a test for trend across disease progression in the percentages of cells with more than 2 signals per nucleus within each case.

### RESULTS

Representative examples of the histopathological entities are shown in Figure 1. Hyperplasia was either mild or moderate. The

© 2000 Cancer Research Campaign

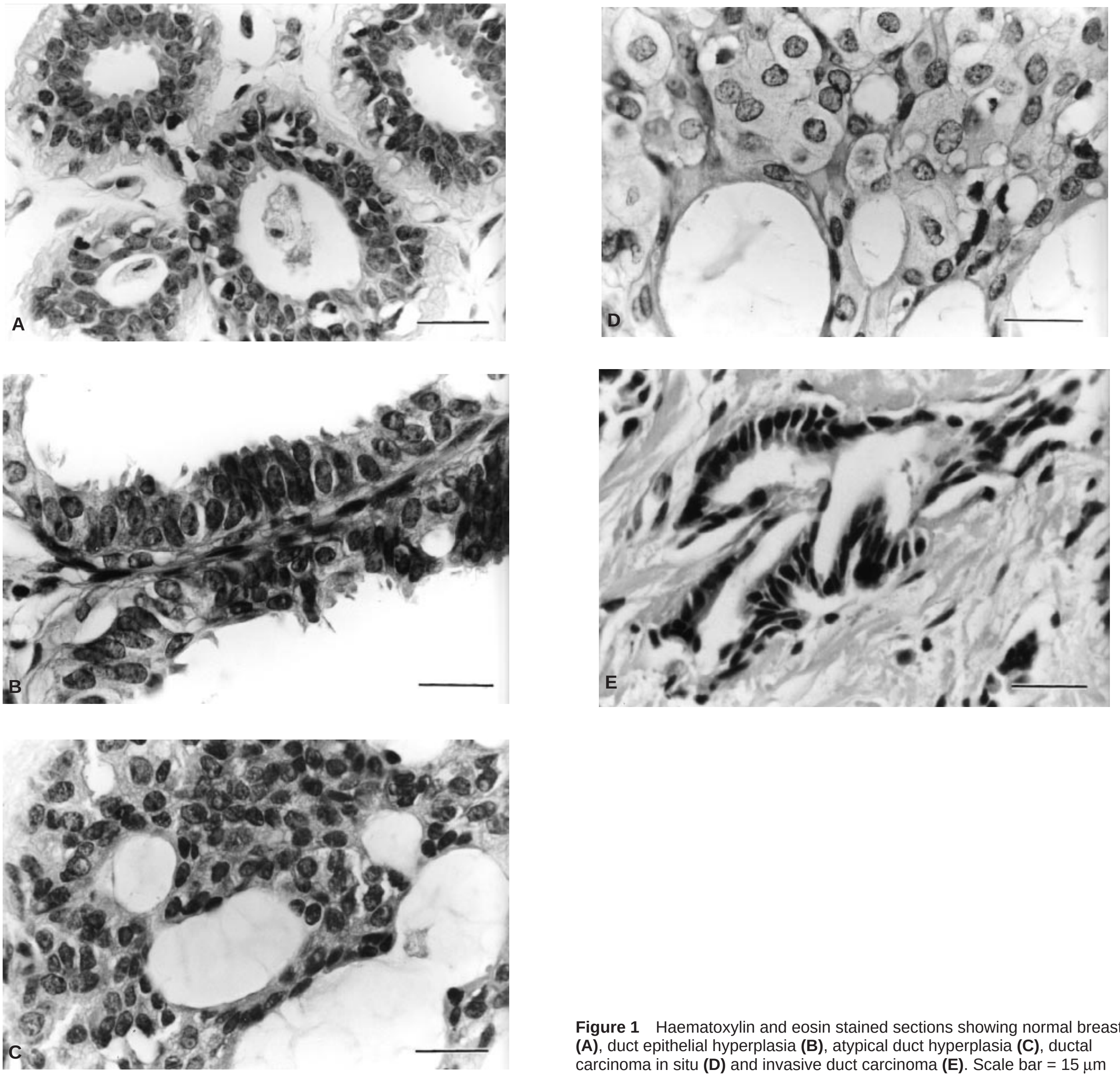

**Figure 1** Haematoxylin and eosin stained sections showing normal breast **(A)**, duct epithelial hyperplasia **(B)**, atypical duct hyperplasia **(C)**, ductal carcinoma in situ **(D)** and invasive duct carcinoma **(E)**. Scale bar = 15 μm

**Table 1** Number of microscopic fields and cells examined by FISH

| | | Normal | | Hyperplasia | | ADH | | DCIS | | Invasive cancer | |
|---|---|---|---|---|---|---|---|---|---|---|---|
| **Case no.** | **No. of blocks** | **Fields** | **Cells** | **Fields** | **Cells** | **Fields** | **Cells** | **Fields** | **Cells** | **Fields** | **Cells** |
| 1 | 1 | 5 | 239 | 7 | 424 | | | | | | |
| 2 | 3 | 14 | 801 | 10 | 512 | 7 | 356 | 10 | 600 | 10 | 516 |
| 3 | 3 | 16 | 398 | 6 | 231 | 14 | 819 | | | 5 | 271 |
| 4 | 3 | 14 | 624 | 14 | 878 | | | 6 | 158 | 10 | 244 |
| 5 | 3 | 13 | 830 | 8 | 403 | 6 | 406 | 8 | 256 | 7 | 149 |
| 6 | 2 | 13 | 888 | 14 | 814 | 6 | 426 | | | | |
| 7 | 2 | 12 | 549 | 6 | 168 | 6 | 407 | | | | |
| Total | 17 | 87 | 4329 | 65 | 3430 | 39 | 2414 | 24 | 1014 | 32 | 1180 |
| Average | | | 618 | | 490 | | 482 | | 338 | | 295 |

 *© 2000 Cancer Research Campaign*

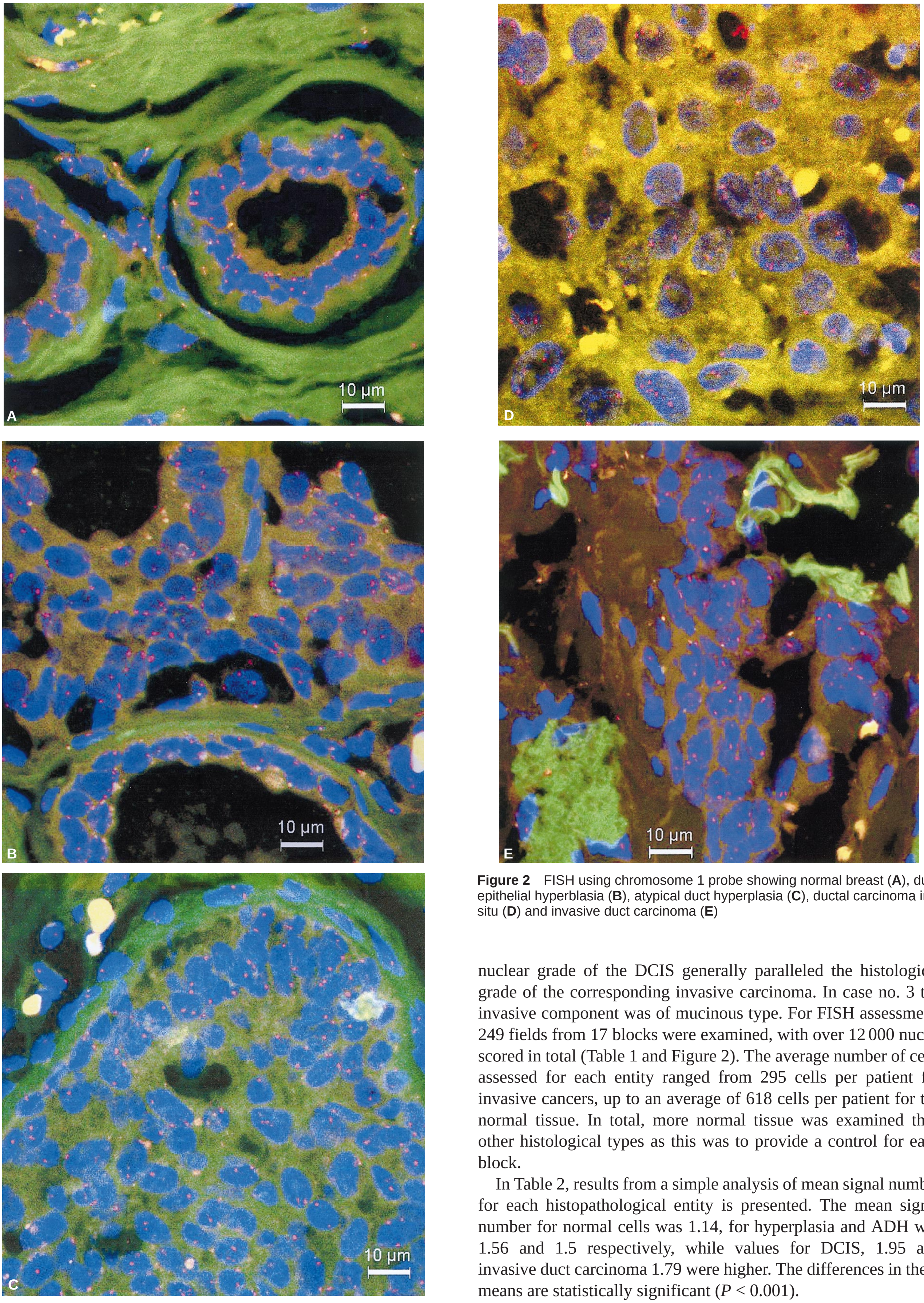

**Figure 2** FISH using chromosome 1 probe showing normal breast (**A**), duct epithelial hyperblasia (**B**), atypical duct hyperplasia (**C**), ductal carcinoma in situ (**D**) and invasive duct carcinoma (**E**)

nuclear grade of the DCIS generally paralleled the histological grade of the corresponding invasive carcinoma. In case no. 3 the invasive component was of mucinous type. For FISH assessment, 249 fields from 17 blocks were examined, with over 12 000 nuclei scored in total (Table 1 and Figure 2). The average number of cells assessed for each entity ranged from 295 cells per patient for invasive cancers, up to an average of 618 cells per patient for the normal tissue. In total, more normal tissue was examined than other histological types as this was to provide a control for each block.

In Table 2, results from a simple analysis of mean signal number for each histopathological entity is presented. The mean signal number for normal cells was 1.14, for hyperplasia and ADH was 1.56 and 1.5 respectively, while values for DCIS, 1.95 and invasive duct carcinoma 1.79 were higher. The differences in these means are statistically significant ($P < 0.001$).

*© 2000 Cancer Research Campaign*

**Table 2** Analysis of mean signal number

| Diagnosis | No. | Mean | Standard deviation | Standard error | 95% Confidence interval for mean | |
|---|---|---|---|---|---|---|
| | | | | | Lower bound | Upper bound |
| Normal | 17 | 1.1361 | 0.1949 | 0.04727 | 1.0359 | 1.2363 |
| Hyperplasia | 9 | 1.5608 | 0.2874 | 0.09581 | 1.3399 | 1.7818 |
| ADH | 6 | 1.4992 | 0.4252 | 0.1736 | 1.0529 | 1.9454 |
| DCIS | 3 | 1.9499 | 0.4838 | 0.2793 | 0.7480 | 3.1518 |
| Invasive | 4 | 1.7944 | 0.5546 | 0.2773 | 0.9119 | 2.6868 |
| Total | 39 | 1.4201 | 0.4153 | 0.06650 | 1.2855 | 1.5547 |

To control for possible errors introduced by variations in section thickness the mean signal number for each entity was normalized relative to a mean signal number of one for the normal tissue (Table 3). The results were not meaningfully different from those presented in Table 2. Thus it was considered that variation in section thickness had a negligible influence on the mean number of signals detected.

The average number of signals for each entity within each case is presented in Figure 3. Using the criterion for monosomy of significantly fewer signals in lesional tissue than in the control normal tissue from that same block, we were not able to demonstrate monosomy in any of the cases. As suggested by Tables 2 and 3, values for hyperplasia and ADH, and those for DCIS and invasive carcinoma each appear to cluster together in pairs.

The percentage of cells containing greater than 2 signals per nucleus for each case is presented in Table 4. A few normal cells had more than 2 signals per nucleus. Six of the seven cases showed a significant trend towards an increasing proportion of cells with more than 2 signals per nucleus with increasing histological progression ($P < 0.001$). In case 3, no such trend was seen.

### DISCUSSION

Benign proliferative disease of the breast is very common and only a very small proportion of affected women go on to develop invasive carcinoma. Certain histopathological features indicate the overall degree of relative risk for this progression and these are based largely on epidemiological studies. However, histopathological

**Table 3** 'Normalized' mean signal number relative to the mean number in the normal tissue

| | No. | Mean | Standard deviation | Standard error | 95% Confidence interval for mean | | Minimum | Maximum |
|---|---|---|---|---|---|---|---|---|
| | | | | | Lower bound | Upper bound | | |
| Hyperplasia | 9 | 1.362 | 0.1452 | 0.0489 | 1.2504 | 1.4736 | 1.12 | 1.56 |
| ADH | 6 | 1.349 | 0.1789 | 0.0730 | 1.1605 | 1.5369 | 1.18 | 1.56 |
| DCIS | 3 | 1.651 | 0.2839 | 0.1639 | 0.9465 | 2.3569 | 1.37 | 1.94 |
| IDC | 4 | 1.646 | 0.7158 | 0.3579 | 0.5075 | 2.7856 | 0.86 | 2.53 |
| Total | 22 | 1.449 | 0.3405 | 0.07259 | 1.2988 | 1.6007 | 0.86 | 2.53 |

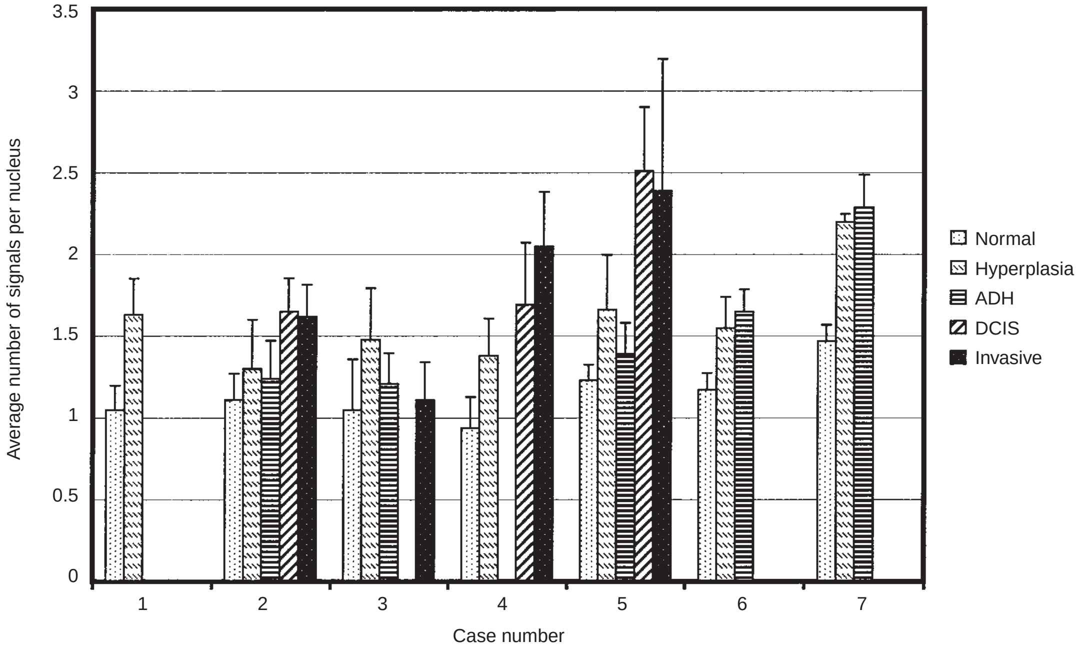

**Figure 3** Average signal number per entity within each case. (Error bars = standard error of the mean)

*© 2000 Cancer Research Campaign*

**Table 4** Percentage of cells with more than two signals per nucleus

| | Normal | Hyperplasia | ADH | DCIS | Invasive | Chi-squared trend | *P*-value |
|---|---|---|---|---|---|---|---|
| 1 | 1.26 | 16.51 | | | | 36.3 | < 0.001 |
| 2 | 1.87 | 8.20 | 7.30 | 17.83 | 10.66 | 69.8 | < 0.001 |
| 3 | 1.51 | 6.06 | 4.86 | | 1.48 | 0.07 | = 0.79 |
| 4 | 0.48 | 3.76 | | 17.72 | 27.46 | 234 | < 0.001 |
| 5 | 1.93 | 14.14 | 6.90 | 48.83 | 41.61 | 331.4 | < 0.001 |
| 6 | 1.69 | 9.46 | 12.21 | | | 60.9 | < 0.001 |
| 7 | 3.99 | 33.93 | 39.56 | | | 189.4 | < 0.001 |

diagnoses are subject to significant interobserver variation. Also, how closely histopathological progression accurately reflects biological disease progression is unclear. Grade 1 (well-differentiated) invasive cancers may arise from low grade DCIS, rather than necessarily requiring a progression through intermediate and high grade DCIS before invasion occurs (Lakhani, 1999). In other cases, extensive high-grade DCIS may be present with minimal or no invasive disease. Better knowledge of the underlying biology of proliferative breast disease and how it relates to both in situ and invasive cancer may allow more accurate predictions about which patients require earlier treatment intervention.

Some of the chromosomal abnormalities commonly demonstrated in invasive breast cancers have also been demonstrated in benign breast disease. Dietrich et al (1995) using short-term cultures demonstrated chromosomal abnormalities in 31 of 45 cases of benign proliferative breast disease. Loss of heterozygosity (LOH) was demonstrated in 15% of cases of hyperplasia of usual type (O'Connell et al, 1994). Using microdissection, LOH was demonstrated in 5/9 informative cases of ADH on chromosome 16q and on 2/8 informative cases on chromosome 17p (Lakhani et al, 1995). LOH was also demonstrated in 0–13% of up to 23 cases of hyperplasia, including loci at 17q, 17p and 16q (Lakhani et al, 1996).

Micale et al (1994) investigated chromosomal aneusomy in both proliferative and malignant lesions of the breast, using pericentromeric probes for FISH analysis on paraffin sections. Loss of chromosome 1 was not identified in any case and gain was not identified in any of the proliferative lesions but only in cases of DCIS and invasive carcinoma. Visscher et al (1996) also studied examples of both ductal and lobular carcinoma in situ together with a range of proliferative breast lesions, using FISH on paraffin sections. While 70% of the DCIS cases showed chromosomal aneuploidy (including five of five patients who had concurrent invasive disease) none of the proliferative lesions showed any detectable chromosomal gains.

Chromosome 1 aneusomy however, has been demonstrated in some examples of benign breast disease, including those with near diploid DNA content (Verdoodt et al, 1994). A total of 8.6% of nuclei from benign cases displayed more than 2 signals per nucleus compared with 7% of nuclei from normal tissue. The mean number of signals per nucleus was significantly different between the benign cases and the ductal carcinomas, but not between the nonductal carcinomas (lobular and special types) and benign disease. The mean number of signals for the benign cases was 1.88–2.13, while for the carcinomas it ranged from 1.49 to 3.69.

Unlike Micale et al (1994) and Visscher et al (1996), but more in keeping with the findings of Verdoodt et al (1994), we demonstrated increased chromosome 1 copy number in hyperplasia as well as in in situ and invasive malignancy. Due to truncation of nuclei in paraffin sections, signal number under-representation was consistently present, however, also assessing normal tissue within each section allowed comparisons between the different entities to be made. Compared with normal tissue, the mean signal number for each entity overall was elevated. Interestingly, the values for hyperplasia and ADH clustered together, as did those for DCIS and invasive ductal carcinoma, rather than being evenly positioned along a spectrum. When the mean signal number for each entity was 'normalized' to a mean signal number of one for normal tissue, this clustering of signal numbers was still evident. While the sample number is relatively small, this implies that the risk for the later development of invasive carcinoma for ADH is similar to that for hyperplasia, at least in terms of the contribution of chromosome 1. The biological contribution of increased chromosome 1 copy number for in situ and invasive disease also appeared similar, again implying that other genetic events are important overall in defining an invasive phenotype.

In each case, except case 3, progression of signal number was seen in parallel with histopathological progression. The sample studied though may have been biased in that the cases were selected to include a range of histological lesions. Chromosomal abnormalities in the benign components of this study may have been more frequent than in a group of patients who have hyperplasia alone. The invasive component of case 3 had a mucinous phenotype, possibly reflecting a different biological basis.

Some variation in the average number of signals per nucleus was seen between cases. A number of factors may explain this. The length of time a specimen is in formalin affects the amount of tissue shrinkage that occurs, therefore affecting nuclear size and the number of signals present in a section. The degree of nuclear enlargement both in benign and malignant tissues may vary both within and between cases. The specific chromosomal abnormalities underlying the increased copy number seen here most probably varied from case to case, again affecting the number of signals detected. While chromosome 1 abnormalities are very common in breast cancer, a range of mechanisms underlies this (Heim and Mitelman, 1995).

Signal distribution was also used to assess signal count. Detecting occasional normal cells with more than 2 signals per nucleus probably represents misinterpretation of overlapping nuclei. Again, except for case 3, where no significant trend was

© 2000 Cancer Research Campaign seen, in each of the other cases a significant trend was identified within each case of an increasing percentage of cells with greater than 2 signals per nucleus occurring in parallel with histopathological progression.

In summary, the results here support the concept that benign proliferative breast disease is a biological precursor of in-situ and invasive ductal cancer and that increased chromosome 1 copy number is an early part of this process. The clustering of mean signal counts for ADH with those for hyperplasia suggests in terms of chromosome 1 that although an increased risk of invasive malignancy is present, that ADH will not necessarily progress. This is in keeping with epidemiological studies in which ADH gives an increased risk for later invasive disease but anywhere in either breast rather than at the original site at which the ADH occurred. Perhaps these early changes indicate a field effect with the accumulation of further genetic changes required for the later development of in situ or invasive disease.

### ACKNOWLEDGEMENTS

This work was supported by the Wilhelm Sander-Stiftung (96.070.1), Munich, Germany. Margaret Cummings was supported by the Deutscher Akademischer Austauschdienst. We gratefully acknowedge the excellent technical assistance given by Daniela Angermeier and Ernst Mannweiler.

### REFERENCES

Aubele M, Zitzelsberger H, Szucs S, Werner M, Braselmann H, Hutzler P, Rodenacker K, Lehmann L, Minkus G and Hofler H (1997) Comparative FISH analysis of numerical chromosome 7 abnormalities in 5-µm and 15-µm paraffin-embedded tissue sections from prostatic carcinoma. *Histochem Cell Biol* **107**: 121–126

Buckley CH, Butler EB and Fox H (1982) Cervical intraepithelial neoplasia. *J Clin Pathol* **35**: 1–13

Dietrich CU, Pandis N, Teixeira MR, Bardi G, Gerdes AM, Andersen JA and Heim S (1995) Chromosome abnormalities in benign hyperproliferative disorders of epithelial and stromal breast tissue. *Int J Cancer* **60**: 49–53

Elston CW (1987) Grading of invasive carcinoma of the breast. In: *Diagnostic Histopathology of the Breast*, Page DL and Anderson JJ (eds), pp. 399–311. Churchill Livingstone: Edinburgh

Fox H and Buckley CH (1982) The endometrial hyperplasias and their relationship to endometrial neoplasia. *Histopathology* **6**: 493–510

Harrison M, Magee HM, O'Loughlin J, Gorey TF and Dervan PA (1995) Chromosome 1 aneusomy, identified by interphase cytogenetics, in mammographically detected ductal carcinoma in situ of the breast. *J Pathol* **175**: 303–309

Harrison MM, Magee HM, O'Loughlin J, Gorey TF, Coyne JD and Dervan PA (1997) Comparison of chromosome 1 aneusomy detected by interphase cytogenetics and DNA ploidy in carcinoma of the breast. *Histopathology* **30**: 221–226

Heim S and Mitelman F (1995) *Cancer Cytogenetics* 2nd edn. Wiley-Liss: New York

Lakhani SR (1999) The transition from hyperplasia to invasive carcinoma of the breast. *J Pathol* **187**: 272–278

Lakhani SR, Collins N, Stratton MR and Sloane JP (1995) Atypical ductal hyperplasia of the breast: clonal proliferation with loss of heterozygosity on chromosomes 16q and 17p. *J Clin Pathol* **48**: 611–615

Lakhani SR, Slack DN, Hamoudi RA, Collins N, Stratton MR and Sloane JP (1996) Detection of allelic imbalance indicates that a proportion of mammary hyperplasia of usual type are clonal, neoplastic proliferations. *Lab Invest* **74**: 129–135

Mantel N and Haenszel W (1959) Statistical aspects of the analysis of data from retrospective studies of disease. *J Natl Cancer Inst* **22**: 719–748

Micale MA, Visscher DW, Gulin SE and Wolman SR (1994) Chromosomal aneuploidy in proliferative breast disease. *Hum Pathol* **25**: 29–35

O'Connell P, Pekkel V, Fuqua S, Osborne CK and Allred DC (1994) Molecular-genetic studies of early breast-cancer evolution. *Breast Cancer Res Treat* **32**: 5–12

Page DL and Dupont WD (1990) Anatomic markers of human premalignancy and risk of breast cancer. *Cancer* **66**: 1326–1335

Verdoodt B, Castelain P, Bourgain C and Kirsch Volders M (1994) Aneuploidy for chromosome 1 and overall DNA content in benign and malignant breast disease. *Cancer Genet Cytogenet* **78**: 53–63

Visscher DW, Wallis TL and Crissman JD (1996) Evaluation of chromosome aneuploidy in tissue sections of preinvasive breast carcinomas using interphase cytogenetics. *Cancer* **77**: 315–320

Zitzelsberger H, Szucs S, Weier HU, Lehmann L, Braselmann H, Enders S, Schilling A, Breul J, Hofler H and Bauchinger M (1994) Numerical abnormalities of chromosome 7 in human prostate cancer detected by fluorescence in situ hybridization (FISH) on paraffin-embedded tissue sections with centromere-specific DNA probes. *J Pathol* **172**: 325–335

 *© 2000 Cancer Research Campaign*
